# COVID-19 Home Deaths without Medical Assistance in Northeastern Brazil

**DOI:** 10.4269/ajtmh.20-1210

**Published:** 2020-12-10

**Authors:** Pedro Mansueto Melo de Souza, Gunter Gerson, Carlos Eduardo Lopes Soares, Sarlene Gomes de Souza, Josebson Silva Dias, Deborah Nunes de Melo, Erasmo Miessa Ruiz, Fabio Tavora, Luciano Pamplona de Goes Cavalcanti

**Affiliations:** 1 “Dr. Rocha Furtado” Death Verification Service, Fortaleza, Brazil;; 2 Ceara State University, Fortaleza, Brazil;; 3 Ceara Federal University, Fortaleza, Brazil;; 4 Christus University Center, Fortaleza, Brazil

## Abstract

Since its beginning in Wuhan, China, in December 2019, the disease caused by COVID-19 has reached more than 27 million confirmed cases and more than 880 thousand deaths worldwide by early September 2020. Although it is known that some of these deaths may have been influenced by the overload of health systems, the world medical literature lacks data on deaths due to COVID-19 in patients who have not received medical assistance. We conducted a retrospective transversal study to report the clinical and epidemiological profile of the first 200 consecutive cases of home deaths without medical assistance caused by COVID-19 diagnosed by verbal autopsy and real-time PCR in samples of postmortem nasopharyngeal swabs, in the state of Ceara, in Northeastern Brazil. The data show a slightly increased prevalence of cases in males (57%) and an average age of 76.8 years. Previous comorbidities were reported in 85.5% of cases, the most common being cardiovascular disease (45%), neurological disease (30%), and diabetes (29%). The main symptoms reported were dyspnea (79%), fever (75.5%), cough (69%), and fatigue (42.5%). The average time between the onset of illness and death was 7.3 days, being statistically shorter in patients who had previous comorbidities (*P* = 0.0215). This is the first study to evidence the clinical and epidemiological characteristics of COVID-19 home deaths without medical assistance, which may represent a considerable portion of the pandemic burden, especially in the context of health system overload.

## INTRODUCTION

Since its beginning in Wuhan, China, in December 2019, the disease caused by COVID-19 has reached more than 200 countries, and, as of early September 2020, there were more than 27 million confirmed cases and 880 thousand deaths from COVID-19 worldwide, from which four million confirmed cases and 126 thousand deaths occurred only in Brazil.^1^

The most lethal clinical manifestation of COVID-19 is the severe acute respiratory syndrome,^2^ which demands hospitalization and intensive care treatment. Thus, the rapid spread of the disease has led to the overload of health systems, that is, the inability to provide health care in response to growing demand, which occurred even in developed countries such as the Italian region of Lombardy^3^ and the Spanish autonomous region of Madrid,^4^ contributing to the rise in mortality for the insufficient or even the absence of health assistance during the disease.

In the state of Ceara, in Northeastern Brazil, the health system has overloaded only 45 days after the first notified case of COVID-19,^5^ leading to the rise of deaths without medical assistance. Most of these home deaths were investigated by the “Dr. Rocha Furtado” Death Verification Service (SVO-RF) located in the state capital city of Fortaleza, which allowed the execution of this retrospective study to describe the clinical and epidemiological characteristics of patients who died of COVID-19 in their homes, without medical assistance.

Surprisingly, to the best of our knowledge, there are no reports referring to deaths by COVID-19 that lacked medical assistance to this date, underscoring the relevance of this report.

## METHODS

### Participants.

We retrospectively enrolled the first 200 consecutive home deaths by COVID-19 assessed by the SVO-RF, which occurred between March 25 and May 12, 2020, in the state of Ceara, in northeastern Brazil. These patients were all domiciled in the state capital city of Fortaleza and its metropolitan area. The period corresponds to the first phase of the pandemic in the state of Ceara—which peaked in the 19th epidemiological week (from May 3 to 9) and started to decrease from the 20th epidemiological week (from May 10 to 16)—and was chosen for being the most critical period for the health systems when the characteristically exponential growth was presented.^6^

### Deaths without medical assistance.

Deaths that occurred at home, but have received significant medical assistance during the disease that led to death, were not assessed by the SVO-RF, and therefore were not included in this study. For example, patients assisted by family, geriatric, or palliative physicians, when dying at home, have their deaths assessed by their medical doctors. Patients who did not have regular medical assistance but sought emergency assistance during their last hours of life and died in the emergency room had their deaths assessed by the emergency physician, and were also not included in this study. For the same reasons, deaths at nursing homes were not included. Therefore, patients included in this study have not received any medical assistance at all or have received some limited medical assistance like emergency consultations, but not during the dying process.

### Assessment of death by COVID-19.

During the COVID-19 pandemic, the SVO-RF followed the Brazilian Ministry of Health’s guideline^7^ to suspend complete diagnostic autopsies in confirmed or suspected cases of COVID-19. Thus, during the pandemic, SVO-RF assessed the causes of deaths using verbal autopsy and, in selected cases, collecting noninvasive biological samples. The SVO-RF verbal autopsy protocol consists of an interview with the closest relatives regarding previously known comorbidities, signs, symptoms, and chronological sequence of events that lead to death, as most of the verbal autopsy protocols, but it differs from them for having experienced physicians not only to validate the cause of death but also to execute the interview and the external physical examination of the body,^8^ which improves the completeness of the medical records.

Cases were considered suspected for COVID-19 if the deceased presented flu-like symptoms or had close contact with another suspected or confirmed case of COVID-19. For suspected cases, postmortem nasopharyngeal swabs were collected and sent to the Central Laboratory of Public Health of Ceara for real-time PCR study, based on the Charité protocol. Standard laboratory protocols and complete individual protection equipment were used for both workers’ safety and for avoiding contamination of the samples, procedures that were already performed as part of the routine of SVO-RF. If the study resulted in the detection of SARS-CoV-2 genetic material, the case was considered a confirmed death due to COVID-19.

### Data analysis.

The SVO-RF medical records were reviewed for socio-epidemiological (gender, age, race, education level, occupation, and marital status) and clinical variables (symptoms, comorbidities, and time from illness onset to death). Estimation of any health measures during the disease, including self-medication or alternative medicines, was attempted, but the information was most often incomplete or conflicting because of the lack of medical records and because of the poor socio-educational level of the family of the deceased. Therefore, this variable could not be analyzed.

The clinical and epidemiological characteristics between genders were compared with the Student’s *t* and chi-square tests. The variable “time from illness onset to death” was analyzed as a clinical outcome, and its correlations with other epidemiological and clinical characteristics were assessed with Pearson’s linear regression and Student’s *t*-tests. *P*-value lower than 0.05 was considered statistically significant.

### Ethics.

This study was approved by the Research Ethics Office of the School of Public Health of Ceara (approval number: 33464720.0.0000.5037) and followed the international ethics guidelines for human research rigorously.

## RESULTS

The epidemiological and clinical characteristics of all 200 patients who died due to COVID-19 at home, without medical assistance, are summarized in Table 1.

**Table 1:** Clinical–epidemiologic profile of 200 home deaths by COVID-19 (state of Ceará, Brazil, March 25, 2020–May 12, 2020)

	Total (*n* = 200)		Female (*n* = 86)		Male (*n* = 114)		
	Mean ± SD	Range	Mean ± SD	Range	Mean ± SD	Range	*P*-value*
Age (years)	76.82 ± 14.94	25–101	79.85 ± 14.65	25–101	74.53 ± 14.82	31–100	0.0123†
Race	*n*	%	*n*	%	*n*	%	*P*-value‡
Browns	145	72.5	61	70.9	84	73.7	0.8811
White	50	25.0	23	26.7	27	23.7	
Black	5	2.5	2	2.3	3	2.6	
Education level	*n*	%	*n*	%	*n*	%	*P*-value‡
No formal education	56	28.0	29	33.7	27	23.7	0.2615
Elementary school	107	53.5	45	52.3	62	54.4	
High school	29	14.5	9	10.5	20	17.5	
College	5	2.5	1	1.2	4	3.5	
Ignored	3	1.5	2	2.3	1	0.9	
Occupation	*n*	%	*n*	%	*n*	%	*P*-value‡
Housewives	49	24.5	49†	57.0	0	0.0	< 0.0001†
Farmers	24	12.0	6	7.0	18†	15.8	
Others	127	63.5	31	36.0	96	84.2	
Marital status	*n*	%	*n*	%	*n*	%	*P*-value‡
Single	54	27.0	29	33.7	25	21.9	0.0002†
Married	67	33.5	16	18.6	51†	44.7	
Widowed	69	34.5	39†	45.3	30	26.3	
Divorced	10	5.0	2	2.3	8	7.0	
Symptoms	*n*	%	*n*	%	*n*	%	*P*-value‡
Dyspnea	158	79.0	69	80.2	89	78.1	0.8272
Fever	151	75.5	60	69.8	91	79.8	
Cough	138	69.0	55	64.0	83	72.8	
Fatigue	85	42.5	37	43.0	48	42.1	
Runny nose	30	15.0	13	15.1	17	14.9	
Irritability and confusion	30	15.0	18	20.9	12	10.5	
Headache	24	12.0	11	12.8	13	11.4	
Odynophagia	22	11.0	12	14.0	10	8.8	
Diarrhea	19	9.5	9	10.5	10	8.8	
Myalgia	17	8.5	8	9.3	9	7.9	
Hyporexia	10	5.0	4	4.7	6	5.3	
Nausea and vomiting	10	5.0	6	7.0	4	3.5	
Nasal congestion	4	2.0	2	2.3	2	1.8	
Anosmia	2	1.0	0	0.0	2	1.8	
Other symptoms	16	8.0	7	8.1	9	7.9	
Comorbidities	*n*	%	*n*	%	*n*	%	*P*-value‡
Cardiovascular disease	90	45.0	40	46.5	50	43.9	0.1606
Neurological disease	60	30.0	26	30.2	34	29.8	
Diabetes mellitus	58	29.0	33	38.4	25	21.9	
Respiratory disease	18	9.0	6	7.0	12	10.5	
Psychiatric conditions	11	5.5	5	5.8	6	5.3	
Neoplastic disease	9	4.5	5	5.8	4	3.5	
Kidney disease	8	4.0	3	3.5	5	4.4	
Chronic smoking	31	15.5	11	12.8	20	17.5	
Chronic alcoholism	23	11.5	4	4.7	19	16.7	
Other comorbidities	39	19.5	19	22.1	20	17.5	
Without comorbidities	29	14.5	10	11.6	19	16.7	
	Mean ± SD	Range	Mean ± SD	Range	Mean ± SD	Range	*P*-value*
Days from illness onset to death	7.27 ± 5.18	0–32	7.17 ± 5.21	0–32	7.34 ± 5.18	0–30	0.8215

* Pearson’s linear correlation test.

† Statistically significant.

‡ Student’s *t*-test.

Of these, 114 patients (57%) were male and 86 (43%) were female. The mean age of the patients was 76.82 ± 14.94 years, and the range of age varied from 25 to 101 years. The distribution of cases by age showed a peak between 70 and 79 years, with 64 cases (32.0%). The age of deceased male patients (74.53 ± 14.82 years) was statistically younger than females (79.85 ± 14.65 years; *P* = 0.0123).

The distribution of cases by race showed a prevalence of cases in browns (145 patients, 72.5%), followed by white (50 patients, 25%) and black (five patients, 2.5%). As for the education level, 56 patients (28.0%) had no formal education, 107 (53.5%) had only elementary education, 29 (14.5%) had completed high school, and only five (2.5%) had completed a college degree. Three cases (1.5%) had their education levels ignored by family. There was no statistical difference between males and females regarding race or education level.

The occupation status showed a high amount of different professions, with more than 56 listed. Only two occupations were cited more than 12 times: housewives and farmers, both presenting statistical differences between genders. All the 49 housewives were females and represented 57.0% of all deceased women in this study. There were 24 farmers, with 18 of them being males and six females. Farmers were associated with a statistically shorter period from illness onset to death (see in the following text).

When comparing marital status and genders, there was a statistically higher prevalence in widow among women (45.3%) and married among men (44.7%), without a difference in the proportion of single and divorced patients.

Table 1 also shows the clinical profile of the 200 patients. The main presenting symptoms were dyspnea, reported in 158 patients (79%); fever, reported in 151 (75.5%); cough, reported in 138 (69.0%); and adynamia, reported in 85 patients (42.5%).

Of the total number of cases, 171 patients (85.5%) had at least one comorbidity, whereas 29 patients (14.5%) had no comorbidity. The most common comorbidities were cardiovascular diseases, present in 90 cases (45.0%), followed by neurological diseases in 60 cases (30.0%), diabetes in 58 cases (29.0%), respiratory diseases in 18 cases (9.0%), psychiatric conditions in 11 cases (5.5%), neoplastic diseases in nine cases (4.5%), kidney diseases in eight cases (4.0%), and other less frequent comorbidities were reported in 39 cases (19.5%). Chronic smoking was reported in 31 cases (15.5%) and chronic alcoholism in 23 (11.5%).

The mean number of days from onset of symptoms to death was 7.27 ± 5.18 days, with the range varying from 0 to 32 days, and a median of 6 days. There was no statistical difference between genders regarding symptoms, comorbidities, and time from illness onset to death.

Considering “time from illness onset to death” as our clinical outcome, its correlations with the other clinical and epidemiological variables were assessed and summarized in Table 2. Variables with small frequencies were excluded from this analysis because they did not fulfill parametric requirements for the Student’s *t*-test.

**Table 2:** Association between days from illness onset to death and clinical–epidemiologic characteristics of 200 home deaths by COVID-19 (state of Ceará, Brazil, March 25, 2020–May 12, 2020).

	Days from illness to death		
	Mean ± SD		*P*-value*
Age (years)	7.27 ± 5.18		0.508
Gender	Female	Male	*P*-value†
	7.17 ± 5.21	7.34 ± 5.18	0.8215
Race	White	Nonwhite	*P*-value†
	6.50 ± 4.18	7.53 ± 5.47	0.2261
Education level	Presence	Absence	*P*-value†
No formal education	6.75 ± 5.42	7.47 ± 5.09	0.3777
Elementary school	7.85 ± 5.46	6.60 ± 4.79	0.0894
High school	6.86 ± 3.82	7.34 ± 5.39	0.6479
Occupation	Presence	Absence	*P*-value†
Housewives	7.49 ± 5.88	7.20 ± 4.96	0.7336
Farmers	5.17 ± 4.17	7.56 ± 5.25	0.0338‡
Marital status	Presence	Absence	*P*-value†
Single	7.57 ± 5.71	7.16 ± 4.99	0.6151
Married	7.67 ± 5.19	7.07 ± 5.19	0.4381
Widowed	6.59 ± 4.21	7.67 ± 5.61	0.1815
Symptoms	Presence	Absence	*P*-value†
Dyspnea	7.35 ± 5.34	6.95 ± 4.61	0.6562
Fever	6.94 ± 4.44	8.29 ± 6.97	0.1147
Cough	7.67 ± 5.42	6.37 ± 4.53	0.1003
Fatigue	7.06 ± 4.94	7.43 ± 5.37	0.6216
Runny nose	8.03 ± 5.59	7.14 ± 5.12	0.3830
Irritability and confusion	7.00 ± 4.85	7.32 ± 5.25	0.7579
Headache	8.46 ± 5.44	7.11 ± 5.14	0.2322
Odynophagia	8.23 ± 7.39	7.15 ± 4.86	0.3599
Diarrhea	6.47 ± 2.95	7.35 ± 5.36	0.4829
Myalgia	5.71 ± 3.06	7.42 ± 5.32	0.1941
Other symptoms	7.81 ± 6.09	7.22 ± 5.11	0.6636
Comorbidities	Presence	Absence	*P*-value†
Cardiovascular disease	7.34 ± 5.41	7.21 ± 5.02	0.8548
Neurological disease	6.32 ± 3.87	7.68 ± 5.62	0.0887
Diabetes mellitus	7.36 ± 5.67	7.23 ± 4.99	0.8729
Respiratory disease	7.44 ± 3.38	7.25 ± 5.34	0.8815
Chronic smoking	8.42 ± 5.26	7.06 ± 5.16	0.1799
Chronic alcoholism	8.48 ± 7.45	7.11 ± 4.82	0.2357
Some comorbidity	6.92 ± 4.98	9.31 ± 5.94	0.0215‡

* Pearson’s linear correlation test.

† Student’s *t*-test.

‡ Statistically significant.

There was no statistical significance in the association between the clinical outcome and age, gender, race, education level, or marital status. However, our data showed a slightly shorter period from onset to death in female, white, widowed, and people with no formal education.

Regarding occupation, there was a statistically significant shorter period from illness onset to death among farmers, with 5.17 ± 4.17 days, comparing with 7.56 ± 5.25 days among non-farmers (*P* = 0.0338).

There was no statistical significance when comparing the clinical outcome with the presence of each of the major symptoms, and the presence of each of the major comorbidities. However, when comparing the group of deceased people with some comorbidity versus the group without any comorbidity, a statistically significant difference is seen, with the mean days from illness onset to death of 6.92 ± 4.98 days in deceased people with comorbidities and 9.31 ± 5.94 days in people without comorbidities (*P* = 0.0215).

## DISCUSSION

“Time from illness onset to death” is an important clinical outcome, once a shorter evolution to death implies a more severe disease presentation and, therefore, can be compared with severity and mortality rates. Also, in the context of deaths without medical assistance, it is important to know which groups are more vulnerable and should receive priority health assistance.

According to the current literature, COVID-19 severity and mortality rates are higher among older patients.^9–12^ In one of the first national death reports, there was a higher prevalence in men (61.1%) and patients older than 70 years (62.9%) among the first 54 fatal cases of COVID-19 in South Korea.^13^ In this current study, we found that the average age of the 200 patients was 76.82 years, with a peak incidence between 70 and 79 years, and a male predominance of 57% of cases, gender whom also presented a statistically younger age in the moment of death, when compared with women (*P* = 0.0123).

The prevalence of races among home deaths analyzed in our study (72.5% of brown, 25.0% of white, and 2.5% of black) was similar to the prevalence of the general population in the state of Ceara (66.2%, 27.2%, and 5.9%, respectively).^14^ There was a slightly shorter period of illness onset to death in the white population than in the non-white deceased people with COVID-19, but with no statistical significance. By contrast, recent studies in the United States^15,16^ and in Brazil^17^ showed a higher risk of mortality among nonwhite patients with COVID-19 with medical assistance.

The educational level survey demonstrates the vulnerability of the analyzed population, with 28.0% with no formal education and 53.5% with only elementary school, which may have contributed to the inaccessibility of health systems along with the saturation of the health facilities by the pandemic itself.

Our study identified a statistically significant shorter period from illness onset to death among farmers, contrasting with the absence of reports relating farmers with increased risk for mortality by COVID-19. Only a single study^18^ evidenced similar occupations, such as animal slaughtering and processing industry, as being classified as essential industries during the pandemics and being more exposed to infections.

Concerning the symptoms of COVID-19, one of the first reports from China^19^ evidenced as the most commonly reported symptom fever (71.4%), cough (60.4%), and fatigue (43.9%). A large systematic review and meta-analysis^12^ showed similar results: fever (78.5%), cough (53.8%), and fatigue (25.0%). However, another systematic review^20^ stated that dyspnea was the most significant symptom associated with lethal disease, and the mode of death was predominantly through respiratory or heart failure, which aligns with our findings that evidenced that the most common symptoms associated with home deaths were dyspnea (79.0%), followed by fever (75.5%), cough (69.0%), and fatigue (42.5%).

Among all patients diagnosed with COVID-19 analyzed in a large systematic review,^12^ comorbidities were present in 31.0% of the adult patients, with hypertension being the most prevalent, followed by heart failure, diabetes mellitus, and coronary heart disease, and its meta-analysis also revealed that preexisting comorbidities were associated with a higher relative risk (RR) of disease severity (RR = 2.11 [1.02–4.35]; *P* = 0.046) and in-hospital mortality (RR = 1.69 [1.48–1.94]; *P* < 0.001). In addition, a report focused only on the analysis of deceased patients by COVID-19 showed a much higher prevalence of comorbidities (68.2%), especially hypertension (37.6%), diabetes (22.4%), and coronary heart disease (11.8%).^21^ These last numbers are closer to the ones in our study when we focus only in deceased patients who died at home without medical assistance and found that the majority of patients had some previous comorbidity (85.0%), with the most common being cardiovascular diseases (45%), neurological diseases (30%) and diabetes (29.0%). It is reinforced that the presence of previous comorbidities was significantly related to the reduction in the time between illness onset and death in our study.

We conclude that this is a pioneer report not only for evidencing the clinical and epidemiological profile of patients who died because of COVID-19 without medical assistance but also for demonstrating the similarities and particularities against the current known profile of COVID-19 deaths, which are composed exclusively of medical-assisted deaths.

However, there were three major limitations of this study. The first one was the impossibility of conducting a complete diagnostic autopsy (CDA) with a detailed pathological study of the organs. In performing a CDA, one could potentially rule out some of these deaths that might have been caused by other etiologies in patients who were only carriers of the virus and were manifesting mild active disease, and could also allow a better understanding of the pathophysiology of COVID-19.^22–24^

The second limitation is the absence of ancillary examinations and the dependence in oral testimonials of the poor socio-educational level families to collect data for the verbal autopsy, which may have led to the partial missing of some symptoms, comorbidities, and even the non-suspicion of cases with atypical presentation.

The third limitation was the impossibility to address the major reasons for the absence of medical assistance during these COVID-19 home deaths, as this question was not usually asked in the verbal autopsy protocol and was not registered in the medical records. Therefore, subsequent studies are necessary to evaluate quantitatively and qualitatively why these patients did not receive health care before death, with the overload of the health system being our major hypothesis.

Despite its limitations, the death investigation conducted by SVO-RF greatly enhanced the detection of COVID-19 deaths that would certainly be underreported. It also helped understand the clinical and epidemiological characteristics of this vulnerable portion of the society that lacks access to health systems and may represent a considerable portion of the pandemic burden, especially in the context of health system overload.

## References

[1] World Health Organization, 2020 WHO Coronavirus Disease (COVID-19) Dashboard. Geneva, Switzerland: WHO Available at: https://covid19.who.int/. Accessed September 7, 2020.

[2] Huang C 2020 Clinical features of patients infected with 2019 novel coronavirus in Wuhan, China. Lancet 395: 497–506. 31986264 10.1016/S0140-6736(20)30183-5 PMC7159299

[3] Odone A Delmonte D Scognamiglio T Signorelli C, 2020 COVID-19 deaths in Lombardy, Italy: data in context. Lancet Public Health 5: e310. 32339478 10.1016/S2468-2667(20)30099-2 PMC7182509

[4] Condes E Arribas JR; COVID19 MADRID-S.P.P.M. Group, 2020 Impact of COVID-19 on Madrid hospital system. Enferm Infecc Microbiol Clin [Epub ahead of print]. DOI: 10.1016/j.eimc.2020.06.005. 10.1016/j.eimc.2020.06.005 PMC7315960 38620683

[5] Lemos DRQ 2020 Health system collapse 45 days after the detection of COVID-19 in Ceará, Northeast Brazil: a preliminary analysis. Rev Soc Bras Med Trop 53: e20200354. 32638888 10.1590/0037-8682-0354-2020 PMC7341828

[6] de Souza PMM Gerson G Dias JS de Melo DN de Souza SG Ruiz EM Fernandes Tavora FR Cavalcanti LPG, 2020 Validation of verbal autopsy and nasopharyngeal swab collection for the investigation of deaths at home during the COVID-19 pandemics in Brazil. PLoS Negl Trop Dis 14: e0008830. 33147211 10.1371/journal.pntd.0008830 PMC7641351

[7] Brazilian Ministry of Health, 2020 *Manejo de corpos no contexto do novo coronavírus COVID-19*. Available at: https://antigo.saude.gov.br/images/pdf/2020/marco/25/manejo-corpos-coronavirus-versao1-25mar20-rev5.pdf/. Accessed June 29, 2020.

[8] Leitao J 2014 Comparison of physician-certified verbal autopsy with computer-coded verbal autopsy for cause of death assignment in hospitalized patients in low- and middle-income countries: systematic review. BMC Med 12: 22. 24495312 10.1186/1741-7015-12-22 PMC3912516

[9] Zhou F 2020 Clinical course and risk factors for mortality of adult inpatients with COVID-19 in Wuhan, China: a retrospective cohort study. Lancet 395: 1054–1062. 32171076 10.1016/S0140-6736(20)30566-3 PMC7270627

[10] Chilimuri S Sun H Alemam A Mantri N Shehi E Tejada J Yugay A Nayudu SK, 2020 Predictors of mortality in adults admitted with COVID-19: retrospective cohort study from New York city. West J Emerg Med 21: 779–784. 32726241 10.5811/westjem.2020.6.47919 PMC7390589

[11] Du RH 2020 Predictors of mortality for patients with COVID-19 pneumonia caused by SARS-CoV-2: a prospective cohort study. Eur Respir J 55: 2000524. 32269088 10.1183/13993003.00524-2020 PMC7144257

[12] Jutzeler CR 2020 Comorbidities, clinical signs and symptoms, laboratory findings, imaging features, treatment strategies, and outcomes in adult and pediatric patients with COVID-19: a systematic review and meta-analysis. Travel Med Infect Dis 37: 101825. 32763496 10.1016/j.tmaid.2020.101825 PMC7402237

[13] Korean Society of Infectious Diseases, Korea Centers for Disease Control and Prevention, 2020 Analysis on 54 mortality cases of coronavirus disease 2019 in the Republic of Korea from January 19 to March 10, 2020. J Korean Med Sci 35: e132. 32233161 10.3346/jkms.2020.35.e132 PMC7105509

[14] Brazilian Institute of Geography and Statistics, 2020 IBGE pesquisa nacional por amostra de domicilios contínua anual - tabela 6408 - população residente, por sexo e cor ou raça. Available at: https://sidra.ibge.gov.br/tabela/6408/. Accessed September 1, 2020.

[15] Golestaneh L 2020 The association of race and COVID-19 mortality. EClinicalMedicine 25: 100455. 32838233 10.1016/j.eclinm.2020.100455 PMC7361093

[16] Poulson M Geary A Annesi C Alle L Kenzik K Sanchez S Tseng J Dechert T, 2020 National disparities in COVID-19 outcomes between black and white Americans. J Natl Med Assoc [Epub ahead of print]. DOI: 10.1016/j.jnma.2020.07.009. 10.1016/j.jnma.2020.07.009 PMC7413663 32778445

[17] Baqui P Bica I Marra V Ercole A van der Schaar M, 2020 Ethnic and regional variations in hospital mortality from COVID-19 in Brazil: a cross-sectional observational study. Lancet Glob Health 8: e1018–e1026. 32622400 10.1016/S2214-109X(20)30285-0 PMC7332269

[18] Hawkins D, 2020 Differential occupational risk for COVID-19 and other infection exposure according to race and ethnicity. Am J Ind Med 63: 817–820. 32539166 10.1002/ajim.23145 PMC7323065

[19] Qian CG 2020 Epidemiologic and clinical characteristics of 91 hospitalized patients with COVID-19 in Zhejiang, China: a retrospective, multi-centre case series. QJM 113: 474–481. 32181807 10.1093/qjmed/hcaa089 PMC7184349

[20] Keeley P Buchanan D Carolan C Pivodic L Tavabie S Noble S, 2020 Symptom burden and clinical profile of COVID-19 deaths: a rapid systematic review and evidence summary. BMJ Support Palliat Care 10: 381–384. 10.1136/bmjspcare-2020-002368 32467101

[21] Du Y 2020 Clinical features of 85 fatal cases of COVID-19 from Wuhan. A retrospective observational study, Am J Respir Crit Care Med 201: 1372–1379. 32242738 10.1164/rccm.202003-0543OC PMC7258652

[22] Pomara C Volti GL Cappello F, 2020 COVID-19 deaths: are we sure it is pneumonia? Please, autopsy, autopsy, autopsy!. J Clin Med 9: 1259. 10.3390/jcm9051259 PMC7287760 32357503

[23] Salerno M Sessa F Piscopo A Montana A Torrisi M Patanè F Murabito P Volti GL Pomara C, 2020 No autopsies on COVID-19 deaths: a missed opportunity and the lockdown of science. J Clin Med 9: 1472. 10.3390/jcm9051472 PMC7291342 32422983

[24] Al Nemer A, 2020 Histopathologic and autopsy findings in patients diagnosed with coronavirus disease 2019 (COVID 19): what we know so far based on correlation with clinical, morphologic and pathobiological aspects. Adv Anat Pathol 27: 363–370. 32732586 10.1097/PAP.0000000000000276

